# Regional adenosine-induced hypoperfusion without hyperenhancement on LGE-MRI in young HCM patients: comparison to subjects at risk of HCM and healthy volunteers

**DOI:** 10.1186/1532-429X-17-S1-Q51

**Published:** 2015-02-03

**Authors:** Robert Jablonowski, Eva Fernlund, Anthony H Aletras, Henrik Engblom, Einar Heiberg, Petru Liuba, Håkan Arheden, Marcus Carlsson

**Affiliations:** 1 Cardiac MR group Lund, Dept of Clinical Physiology, Lund University, Lund, Sweden; 2 Dept. of Biomedical Engineering, Faculty of Engineering, Lund University, Lund, Sweden; 3 Dept. of Pediatric Cardiology, Lund University Hospital, Lund University, Lund, Sweden

## Background

The relation between hypoperfusion and fibrosis is not completely understood in hypertrophic cardiomyopathy (HCM). One hypothesis is that hypoperfusion is a primary cause of fibrosis. Therefore, the aim of this study was to use cardiac magnetic resonance (CMR) to 1) measure if regional perfusion is decreased in young patients with HCM and in subjects at risk of HCM compared to normal controls and 2) determine if regional myocardial perfusion is decreased in areas with left ventricular hypertrophy (LVH) without detectable fibrosis.

## Methods

Study participants were divided into either 1) HCM patients (wall thickness>13mm or >3SD on z-score with echocardiography and/or fibrosis), 2) HCM-risk subjects (HCM present among first-degree relatives, but without LVH/fibrosis on CMR), and 3) healthy controls.

**Figure 1:**
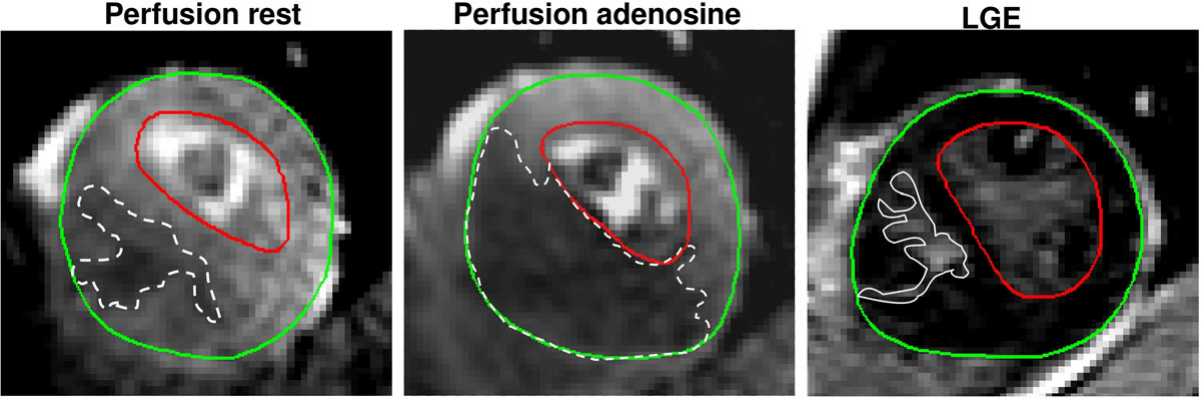
Short-axis perfusion images of the left ventricle at rest (left), adenosine stress (middle) and corresponding late gadolinium enhancement (LGE) image (right) in one representative patient with hypertrophic cardiomyopathy. The extent and severity of ischemic areas (dashed white lines) was larger at adenosine stress compared to rest. The extent was larger for both ischemic areas compared to fibrosis (full white line). Green line=epicardium, red line=endocardium.

Regional perfusion imaging and late gadolinium enhancement (LGE)-CMR was performed on a Philips Achieva 1.5T with a 32-channel coil. Perfusion images were acquired during adenosine (140μg/kg/min) hyperemia and at rest and divided into 16 segments (Figure 1). Maximum upslope (ratio of the perfusion stress and rest values normalized for LV input function) was used for semi-quantitative analysis. Hypertrophic LV segments were analyzed by co-registration with corresponding LGE short-axis images. Differences with p<0.05 were considered statistically significant.

## Results

Twenty-six young subjects were included, seven patients with HCM (22±6 years), eleven subjects at risk of HCM (19±4 years) and eight controls (22±5 years). The LV mass was larger in HCM patients compared to HCM-risk and normal subjects (85±43 vs 46±6, 53±9g/m^2^, p<0.05) and the end-diastolic and end-systolic volumes were smaller for HCM-patients and subjects at risk compared to normal subjects (p<0.05).

The extent and severity of hypoperfused myocardium in HCM patients during adenosine was larger compared to rest and had larger extent compared to fibrosis (Figure 1).

**Figure 2:**
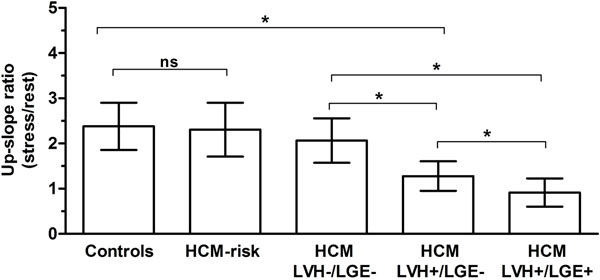
Regional myocardial perfusion expressed as up-slope ratio (mean±SD) in normal controls (n=105 segments), patients at risk of hypertrophic cardiomyopathy (HCM-risk) (n=151 segments) and HCM-patients, subdivided into three groups of segments: (i) without left ventricular hypertrophy (LVH) and fibrosis (LGE) (LVH-/LGE-, n=60 segments), (ii) segments with LVH but without fibrosis (LVH+/LGE-, n=22 segments) and (iii) segments with LVH and fibrosis (LVH+/LGE+, n=22 segments).

Analysis of perfusion showed no significant difference between controls and HCM-risk subjects (2.4±0.5 vs 2.3±0.6, p=0.09) (Figure 2). Segments with hypertrophy and no fibrosis in HCM-patients showed decreased perfusion compared to segments without hypertrophy (1.3±0.3 vs. 2.1±0.5, p<0.0001). Segments with both hypertrophy and fibrosis showed even lower perfusion (0.9±0.3, p<0.0001).

## Conclusions

This study has demonstrated decreased regional perfusion in myocardium with hypertrophy compared to non-hypertrophied myocardium and even lower perfusion in hypertrophy with fibrosis in young HCM patients. Subjects at risk of HCM showed normal regional perfusion during adenosine. The region of hyperemia-induced hypoperfusion has a larger extent than the fibrotic myocardium on LGE. This supports the hypothesis that myocardial hypoperfusion is primary and that fibrosis is caused by replacement fibrosis.

## Funding

Swedish Heart-Lung foundation, Lund University, Region of Scania.

